# Magnetic Graphene-Based Nanosheets with Pluronic F127-Chitosan Biopolymers Encapsulated α-Mangosteen Drugs for Breast Cancer Cells Therapy

**DOI:** 10.3390/polym14153163

**Published:** 2022-08-03

**Authors:** Andri Hardiansyah, Ahmad Randy, Rizna Triana Dewi, Marissa Angelina, Nurfina Yudasari, Sri Rahayu, Ika Maria Ulfah, Faiza Maryani, Yu-Wei Cheng, Ting-Yu Liu

**Affiliations:** 1 Research Center for Advanced Material, National Research and Innovation Agency (BRIN), Tangerang Selatan 15314, Indonesia; srir008@brin.go.id (S.R.); ikam003@brin.go.id (I.M.U.); 2 Research Center for Pharmaceutical Ingredients and Traditional Medicine, National Research and Innovation Agency (BRIN), Tangerang Selatan 15314, Indonesia; ahmad.randy@brin.go.id (A.R.); rizn001@brin.go.id (R.T.D.); mari011@brin.go.id (M.A.); 3 Research Center for Photonics, National Research and Innovation Agency (BRIN), Tangerang Selatan 15314, Indonesia; nurfina.yudasari@brin.go.id; 4 Research Center for Advanced Chemistry, National Research and Innovation Agency (BRIN), Tangerang Selatan 15314, Indonesia; faiz003@brin.go.id; 5 Department of Chemical Engineering, Ming Chi University of Technology, New Taipei City 243303, Taiwan; louischengblue@gmail.com; 6 Department of Materials Engineering, Ming Chi University of Technology, New Taipei City 243303, Taiwan; 7 Research Center for Intelligent Medical Devices, Center for Plasma and Thin Film Technologies, Ming Chi University of Technology, New Taipei City 243303, Taiwan

**Keywords:** graphene oxide, pluronic F127, α-mangosteen, human breast cancer, chitosan

## Abstract

In this study, multifunctional chitosan-pluronic F127 with magnetic reduced graphene oxide (MRGO) nanocomposites were developed through the immobilization of chitosan and an amphiphilic polymer (pluronic F127) onto the MRGO. Physicochemical characterizations and in-vitro cytotoxicity of nanocomposites were investigated through field emission scanning electron microscopy (FESEM), X-ray diffraction (XRD), Fourier-transform infrared (FTIR) spectroscopy, particle size analysis, vibrating sample magnetometer, Raman spectroscopy and resazurin-based in-vitro cytotoxicity assay. FESEM observation shows that the magnetic nanoparticles could tethered on the surface of MRGO, promoting the magnetic properties of the nanocomposites. FTIR identification analysis revealed that the chitosan/pluronic F127 were successfully immobilized on the surface of MRGO. Furthermore, α-mangosteen, as a model of natural drug compound, was successfully encapsulated onto the chitosan/pluronic F127@MRGO nanocomposites. According to in-vitro cytotoxicity assay, α-mangosteen-loaded chitosan/pluronic F127@MRGO nanocomposites could significantly reduce the proliferation of human breast cancer (MFC-7) cells. Eventually, it would be anticipated that the novel α-mangosteen-loaded chitosan/pluronic F127@MRGO nanocomposites could be promoted as a new potential material for magnetically targeting and killing cancer cells.

## 1. Introduction

The discovery and development of novel, safe, and non-toxic anticancer medicines and drug carrier that could reduce toxicity arising from the systemic distribution of anticancer medications in the body has grown in importance in cancer treatment [1]. Nowadays, the clinical chemotherapeutic agents, such as doxorubicin, cyclophosphamide, oxaliplatin or a combination of drugs, exhibit undesirable toxicity and severe side effects [2]. Thus, the discovery and development of anticancer or therapeutic agents with less potent cytotoxicity or no side-effects to the normal cells is needed as an important part of treating cancer or tumor cells. In this respect, a large number of natural compounds have been investigated [3]. *Garcinia mangostana* L. known by the name of mangosteen is a member of the Clusiaceae family and from the genus *Garcinia*. Mangosteen is a tropical fruit plant found in Southeast Asia. Mangosteen peel is widely used in traditional medicine. The polyphenol compound “xanthones” is the main component of mangosteen peel. The dominant xanthones compounds in mangosteen peel are α-mangostin (69.01%), γ-mangostin (17.86%), and followed by other compounds such as gartanin, 8-deoxygartanin, and garcinon. α-mangosteen, a xanthone derivative from the pericarp of Garcinia mangostana L., has been proven to reduce cancer cell survival and proliferation as well as promote apoptosis without accelerating the onset of adverse effects. α-mangosteen has been found to block a number of cell-signaling pathways, including those that control apoptosis, proliferation, invasion, angiogenesis, metastasis, and inflammation. The reported activity of α-mangosteen against a wide variety of cancers reflects its ability to affect multiple targets [4,5]. Herdiana et al. proposed that α-mangostin could inhibits carcinogenesis process including cell proliferation and promoting apoptosis through decreasing the creation of cancerous compounds in breast cancer [6]. 

A wide variety of materials such as polymeric material [7,8,9], dendrimers [10], liposomes [5,11,12,13,14], magnetic nanoparticles [13], or graphene-based materials [15,16,17] have been proposed as a drug carrier to deliver anticancer drugs. Since it can be loaded with various anticancer drugs and can load both hydrophilic and hydrophobic pharmaceutical agents, graphene oxide (GO), a family of graphene-based material derivatives, is one of the potential novel drug delivery systems that is being developed widely. Previous investigations have developed the graphene-based materials as a compartment for drug carrier system. Yaghoubi et al. used GO carboxylation as a drug delivery moiety for doxorubicin and curcumin delivery [18]. Rodrigues et al. synthesized hydrophilic graphene-based yolk-shell magnetic nanoparticles functionalized with copolymer pluronic F127 [19]. Gurunathan et al. developed GO with platinum nanoparticle nanocomposites as therapeutic agents for prostate cancer [20]. Ahamed et al. used GO in combination with silver nanoparticles as anticancer agent for MCF-7 cells. They demonstrated that the killing potential of silver/reduced GO against MCF-7 and lung cancer (A549) cells was two-fold that of pure silver nanoparticles [21]. Alaizeri et al. revealed the enhancement of anticancer efficacy of SnO_2_ nanoparticles by fusion with graphene derivatives [22]. Liang et al. developed polymer-modified magnetic GO nanocomposite as a drug delivery system for chemo-photothermal therapy application [23]. 

Polyethylene glycol (PEG) is a polymer made up of ethylene ether subunits with various molecular weights, branching chain lengths, and functional groups at the end. The covalent or non-covalent attachment of PEG molecules (PEGylation) to the surface of nanomaterials could increase their stability and solubility in physiological fluids, as well as their accumulation in the reticuloendothelial system, extending the half-life of blood circulation and improving pharmacokinetic behavior [24].

Pluronic F127 (PF127) is a block copolymer that consists of hydrophilic poly(ethylene oxide) (PEO) and hydrophobic poly(propylene oxide) (PPO) with PEO-PPO-PEO sequences. PF127 displays low toxicity and high drug loading capabilities suggesting that PF127 have great potential as a drug carrier for drug delivery system. PF127 is widely employed in biomedical applications. For instance, Lee at al. used PF127 to enhance the compatibility of reduced graphene oxide (RGO)-Fe_3_O_4_ [15]. Furthermore, Hu et al. prepared nanohybrid consists of PF127 and graphene nanosheet which exhibits stability and water dispersibility [25]. Li et al. also used PF127 to be assemble with RGO/Fe_3_O_4_ [26]. Ma et al. developed nanocomposite containing GO, PF127 and methylene blue (MB) for combined photothermal therapy and photodynamic therapy [27]. 

Chitosan is a non-toxic, biocompatible, and biodegradable linear co-polymer comprising glucosamine and N-acetyl glucosamine in a (14)-linked natural based polysaccharide with a structure comparable to cellulose. Chitosan is composed of 2-amino-2-deoxy-β-D-glucan combined with glycosidic linkages. The main amine groups provide the polymeric complex unique characteristics that make it suited for numerous biological applications [28,29]. Chitosan molecules show many important biological properties, including antibacterial, antifungal, anticancer, and immunostimulatory properties. In recent years, chitosan molecules have also been employed in transporting drugs and genes, biosensors, wound healing, tissue engineering regeneration, and as carriers of cells and enzymes that have been immobilized. Most classes of bioactive compounds may be targeted and released under regulated conditions using chitosan and its derivative [14,30].

In the present work, chitosan/PF127@MRGO nanocomposite was developed through immobilization of chitosan and PF127 into the surface of MRGO which is simultaneously entrapped a natural anticancer agent, α-mangosteen. To the best of our knowledge, this is the first study to investigate the potential application of biopolymer modified graphene oxide with α-mangosteen encapsulation for human breast cancer (MFC-7) cell lines. Physicochemical characterizations and in vitro cytotoxicity against human breast cancer (MFC-7) cell lines were investigated through field emission scanning electron microscopy (FESEM), X-ray diffraction (XRD), Fourier-transform infrared (FTIR) spectroscopy, particle size analysis, vibrating sample magnetometer, Raman spectroscopy and resazurin-based in-vitro cytotoxicity assay. The potential application of these nanocomposites for cancer treatment would be evaluated. 

## 2. Materials and Methods

### 2.1. Materials

Chitosan (low molecular weight, deacetylated chitin, Poly(D-glucosamine)), PF127, sulfuric acid, phosphoric acid, potassium permanganate, and hydrogen peroxide were purchased from Sigma Aldrich, Burlington, MA, USA. High-purity water purified using a Milli Q Plus water purifier system (Milipore, Burlington, MA, USA), with a resistivity of 18.3 mΩcm was used throughout the experiment. For the cell culture experiments, Dulbecco’s-modified Eagle’s medium (high-glucose DMEM), Dulbecco’s phosphate-buffered saline (DPBS), antibiotic-antimycotic (100×), and fetal bovine serum (FBS) were from Gibco-Thermo Fisher Scientific (Waltham, MA, USA). Alamar Blue Resazurin cell viability reagents were from Thermo Fisher Scientific (Waltham, MA, USA). We used the chemicals of analytical reagent grade as received without further purification. 

In this case, α-mangosteen was isolated from fresh mangosteen peel. Briefly, mangosteen peel was macerated with 96% ethanol for 2 × 24 h. Then the maceration mixture was concentrated and dried to obtain an ethanol extract. The extract was diluted with distilled water and stirred for 1 h, then left overnight (decantation) until a yellow precipitate formed at the bottom of the container. The precipitate formed was dried and further purified by column chromatography to obtain α-mangosteen isolate with a purity > 90% based on HPLC compared to standard α-mangosteen.

### 2.2. Synthesis of GO

GO was developed by using the modified Tour method [31]. Briefly, we used the expandable graphite (EG) and potassium permanganate as the precursor of GO and oxidizing agent, respectively. Phosphoric acid (H_3_PO_4_) was mixed with sulfuric acid (H_2_SO_4_) and subsequently poured into the EG. The mixture was kept stirring on the ice-bath. Furthermore, potassium permanganate was slowly added into the mixture. The reaction was further kept at 50 °C for 12 h. Eventually, we added 400 mL of water and hydrogen peroxide (H_2_O_2_) into the dispersion until the color of GO change from dark brown into yellow solution. The washing and purification process have been carried out into several steps of centrifugation. The final precipitate was harvested and sonicated in water followed by centrifugation at 10,000 rpm for 30 min in order to exfoliate the layers of GO. The final brownish supernatant was considered as GO solution. Meanwhile, the precipitate of un-exfoliated graphene oxide was then removed. The final solution of GO was stored at room temperature prior to characterizations.

### 2.3. Synthesis of Fe_3_O_4_@RGO (MRGO)

Co-precipitation techniques were used with the aim of reducing GO to reduced GO (RGO) and forming a nanocomposite structure of RGO with metal oxides (Fe_3_O_4_), simultaneously. Briefly, an aliquot of GO suspension was mixed homogeneously with FeCl_3_.6H_2_O and FeSO_4_.7H_2_O (molar ratio 2:1) at 80 °C. We added NaOH solution until the pH of the solution reached 10. The product was washed several times and magnetically separated. Eventually, the product was redispersed in water and mixed with PEG in order to enhance the stability and biocompatibility. The final product was termed as magnetic reduced graphene oxide (MRGO). 

### 2.4. Synthesis of Chitosan@MRGO

Briefly, chitosan solution was prepared by mixing chitosan powder in acetic acid solution (2 wt%, 10 mL). Furthermore, we mixed the chitosan solution with the colloidal MRGO formed chitosan@MRGO (Chi@MRGO). Eventually, we separated the Chi@MRGO nanocomposites by decantation with magnetic assistance and washed with DI water to remove impurities. 

### 2.5. Synthesis of Pluronic F127-Chitosan@MRGO

Firstly, PF127 solution was mixed with Chi@MRGO nanocomposite and stirred for 24 h for encapsulation to occur. In order to separate the PF127-Chi@MRGO nanocomposite and eliminate any free PF127, magnetic decantation was used. The mixture was then re-dispersed in DI water. 

### 2.6. Encapsulation Efficiency

First, DMSO was used to dissolve 1 mg/mL of α-mangosteen. Through the use of UV-Vis spectroscopy at a wavelength of 320 nm, a calibration curve for α-mangosteen in DMSO was constructed and quantification was performed [32,33]. A 5 mL of 1 mg/mL α-mangosteen solution was introduced to PF127-Chi@MRGO nanocomposite and bath sonicated for 30 min. To improve the loading of α-mangosteen, we stirred for an additional 60 min. The resultant mixture was magnetically separated, and the supernatant was collected for UV-Vis examination. The absorbance at 320 nm was collected and converted to concentration by using the equation from the calibration graph. Calibration curve was obtained by plotting absorbance of a serial dilution from 15.625 to 62.5 μg mL^−1^ at 320 nm. A linear equation was fitted as absorbance = (0.0331 × concentration) + 0.01352, R^2^ = 0.98. The encapsulation efficiency (was calculated as: EE = total drug-total free drug/total drug × 100%.

### 2.7. Structural and Morphological Characterizations

X-ray powder diffractometer (Rigaku SmartLab X-ray diffractometer, Tokyo, Japan) with Cu Kα radiation of wavelength 0.154 nm was used to observe the structure of nanocomposite. FESEM (JIB-4610F, Tokyo, Japan) was conducted to observe the structure of nanocomposites, respectively. Raman spectroscopy (Horiba iHR320, Kyoto, Japan) was conducted to identify the structure of nanocomposites using Ar-Ne laser source with wavelength of 532 nm. The average particle size of the nanocomposites was determined at 25 °C and pH 7.4 by using dynamic light scattering (DLS) spectrophotometer, Cilas 170 Nano Dual Scattering Particle Size Analyzer, Ariane Group, France with laser diode with wavelength of 638 nm, scattering angle of 90°, and refractive index of 1.33 at 25 °C. 

### 2.8. Cell Culture

The cytotoxicity of nanocomposites was studied on MCF-7 cell (American Type Culture Collection, Manassas, VA, USA. Routine culturing of cells was conducted in DMEM containing 10 vol.% of FBS and 1 vol.% of antibiotic antimycotic solution. Cells were incubated under saturated humid conditions at 37 °C with 5% CO_2_. Medium was changed every two days until reaching approximately 80% confluency.

### 2.9. Cell Cytotoxicity Assay

Cell growth and cytotoxicity were determined using AlamarBlue Resazurin Cell Viability Reagent. In 96 well plates, 5000 cells were seeded in each well and incubated under saturated humid conditions at 37 °C and 5% CO_2_. After 24 h of incubation, the medium was washed with DPBS, and the growth media was changed to a serum-free DMEM. Among these plates, some plates were added with 100 μL of nanocomposites formulations in serum-free DMEM. In addition, the negative control contained only medium, while the positive control was medium containing 5% DMSO. After culturing for 48 h, the cells were washed with DPBS, and the media were replaced with 100 μL of 10% of AlamarBlue reagent in serum-free DMEM. After incubating for another 3 h at 37 °C, the fluorescence signal was observed at 560/590 nm excitation/emission wavelength with a Varioskan Flash multimode reader (Thermo Fisher Scientific, Waltham, MA, USA). The obtained fluorescence signal was subtracted to a fluorescence signal of wells with no cells.

## 3. Results

### 3.1. Formation Mechanism of Nanocomposites

**Figure 1:**
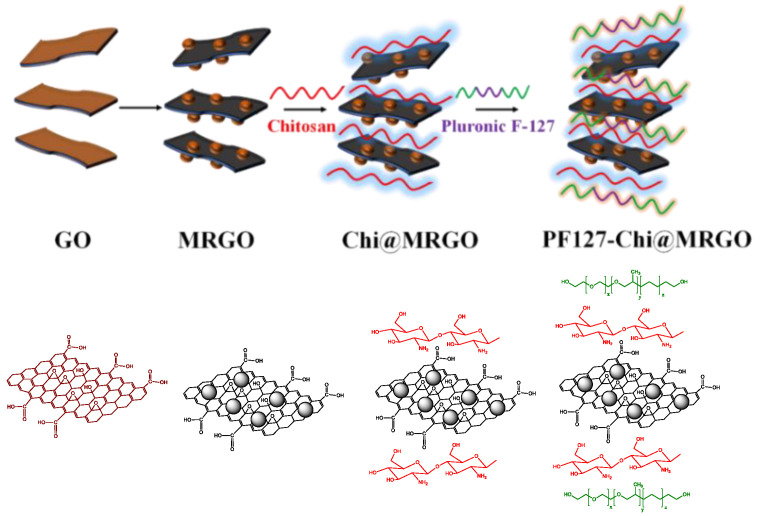
Schematic diagram of nanocomposites formation.

Co-precipitation is a straightforward method to synthesis nanomaterials structures. Co-precipitation reaction was used to develop magnetic nanoparticles that tethered on the surface of GO and reduced the GO simultaneously. Herein, Fe ion can react with oxygen-based functional groups to develop Fe_3_O_4_. This reaction might presence on the surface of GO since GO consist of abundance of oxygen-based functional groups, including carbonyl, carboxyl, epoxy and hydroxyl, thus resulting in MRGO. Moreover, PEG was used to enhance the compatibility of MRGO. Afterwards, we develop the surface modification of MRGO by using chitosan and PF127 to prepare Chi@MRGO and PF127-Chi@MRGO nanocomposites, respectively. Chitosan and PF-127 were used to enhance the biocompatibility of nanocomposites. Eventually, α-mangosteen was loaded into the PF127-Chi@MRGO. In this respect, α-mangosteen could physically be entrapped on the nanocomposites compartment. The schematic diagram of nanocomposite formation is provided in Figure 1.

### 3.2. Structure and Morphological Characterizations

**Figure 2:**
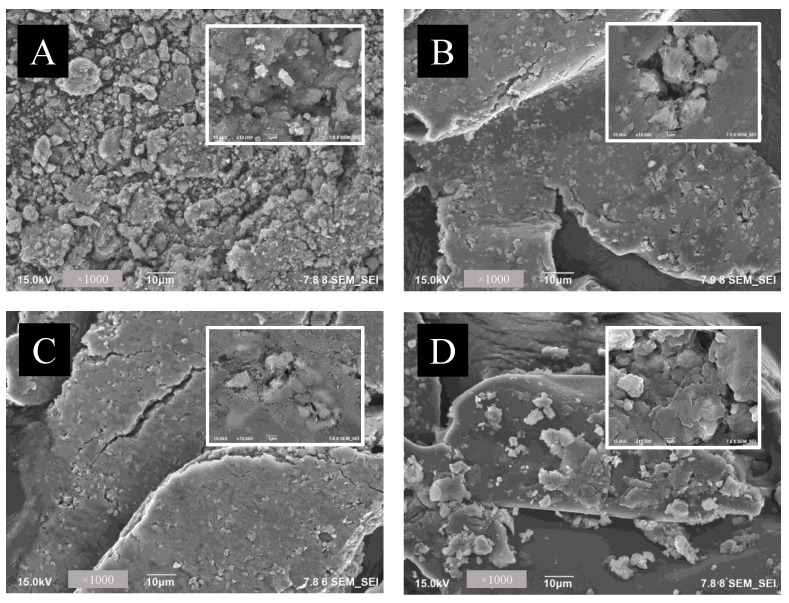
FE-SEM images of MRGO (**A**); Chi@MRGO (**B**); PF127-Chi@MRGO (**C**); α-mangosteen -loaded PF127-Chi@MRGO nanocomposites (**D**).

Figure 2 shows the field emission scanning electron microscopy (FE-SEM) images of nanocomposites. Two types of morphologies were observed including wrinkle structure and nanoparticles structure from RGO sheets and magnetic nanoparticles, respectively (Figure 2A). The FE-SEM images reveal the formation of uniformly distributed Fe_3_O_4_ nanospheres on the surface of reduced graphene oxide (RGO). Similar morphological observation was found for the Chi@MRGO (Figure 2B), PF127-Chi@MRGO (Figure 2C) and α-mangosteen-loaded PF127-Chi@MRGO (Figure 2D).

**Figure 3:**
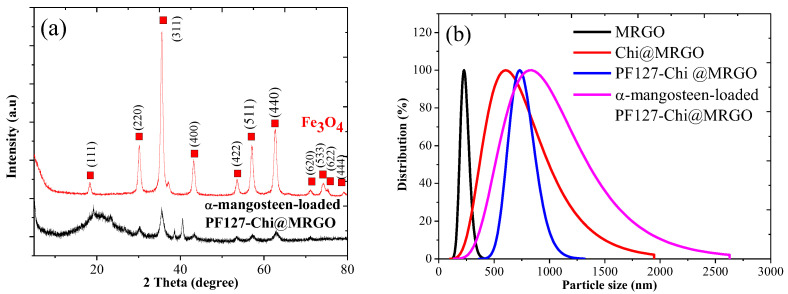
XRD spectra (**a**); particle size distribution (**b**) of nanocomposites.

XRD was used to analyze the structure of nanocomposites. Figure 3a shows that the observed diffraction peaks indicate the GO with a diffraction peak at 2*θ* = 9.44 indexed as the (002) of GO, corresponding to a GO interlayer spacing distance of 0.67 nm, much larger than that of pristine graphite (0.34 nm); and this is caused by loosely stacked GO, due to the abundance of oxygen-based groups and defects caused by the acid oxidation on the surface of GO. The red line in Figure 3a shows that the observed diffraction peaks indicate the magnetite (Fe_3_O_4_) formation with the main peaks at 2*θ* of 18.29 (111), 30.11 (220), 35.48 (311), 36.49 (222), 43.15 (400), 53.71 (422), 57.1 (511), 62.8 (440), 71.19 (620), 74.40 (533), 75.16 (622), and 79.12 (444). The black line in Figure 3a shows the XRD spectra of α-mangosteen -loaded PF127-Chi@MRGO. These spectra indicated that GO was partially reduced, as confirmed by the strong diffraction 2*θ* = 20.54. A minor peak around 20.88° corresponds to the peak of chitosan and PF127 [34,35]. Whilst the peaks around 37° and 42° correspond to the rGO peak [33]. The remaining diffraction peaks in Figure 3a are all assigned to the crystal planes of cubic Fe_3_O_4_ (JCPDS 75-0033), indicating that the Fe^2+^ and Fe^3+^ precursors absorbed on GO were in situ reduced to crystalline Fe_3_O_4_ nanoparticles which were dispersed on the RGO surface. Furthermore, we calculated by using the Scherrer formula that the average size of Fe_3_O_4_ in α-mangosteen-loaded PF127-Chi@MRGO to be around 45 nm. We observed the absence of any other phase in XRD spectra indicating the good purity of magnetic nanoparticles on the surface of RGO. 

The nanocomposite distribution and average particle size were analyzed using The Particle Size Analysis (PSA) technique. Figure 3b. shows the uniformity distribution histogram from nanocomposites. It was found that the particle size increased with the added fillers in the MRGO nanocomposites. The particle size of MRGO, Chi@MRGO, PF127-Chi@MRGO and α-mangosteen-loaded PF127-Chi@MRGO was 232.9, 711.83, 750.33, and 1002.17 nm, respectively. The increasing particle size was due to the attachment of polymeric nanoparticle including chitosan and PF127 on the surface of MRGO and also α-mangosteen loading on the nanocomposites.

**Figure 4:**
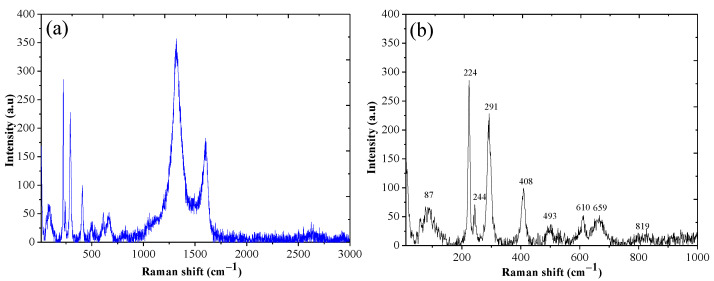
Raman spectra of MRGO (**a**); Magnification of Raman spectra of MRGO (**b**).

Raman spectroscopy was conducted to evaluate the structure of nanocomposites. Figure 4a shows the Raman spectra of MRGO that consists of RGO and Fe_3_O_4_. RGO displays two characteristic band at 1300 and 1500 cm^−1^ corresponding for D and G band, respectively. Figure 4b shows the Raman scattering peaks at that assigned two A_1g_ and three E_g_ Raman modes in a typical Fe_3_O_4_ structure. In addition, MRGO composite shows seven peaks in the Raman spectrum, namely, two A_1g_ modes (225 and 498 cm^−1^) and five E_g_ modes (247, 293, 299, 412, and 613 cm^−1^). We also observed that the presence of unrepaired defects that persisted after the removal of significant quantities of oxygen-containing functional groups is the explanation that the nanocomposites exhibit greater I_D_/I_G_ ratios.

**Figure 5:**
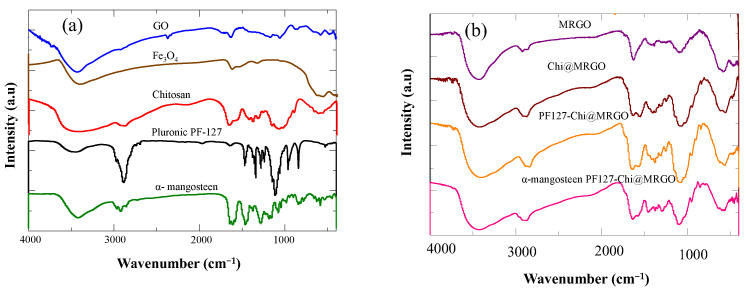
FTIR spectra of GO, Fe_3_O_4_, Chitosan, PF127, and α-mangosteen (**a**); FTIR spectra of MRGO, Chi@MRGO, PF127-Chi@MRGO, and α-mangosteen-loaded PF127-Chi@MRGO nanocomposites (**b**).

The chemical properties and bonding nature of the synthesized nanocomposites was evaluated by FTIR spectroscopy. Figure 5a depicts the FT-IR spectra of GO, Fe_3_O_4_, chitosan, PF127 and α-mangosteen. FTIR spectra of GO shows the peaks at 3435 cm^−1^ corresponds to the O–H vibration, and the peaks at 2926, 1737, 1637, 1383, 1219, and 1054 cm^−1^ corresponds to the C-C, C=O in carboxylic acid and carbonyl functional groups on the edges and basal plane of GO, C=C, C–OH (*v*_C-O-H_), C-O-C (*v*_C-O-H_) and C–O(*v*_C-O_) functional groups, respectively. In the FTIR spectra of Fe_3_O_4_, the peaks at 3413 and 1634 cm^−1^ corresponds to the O–H vibration meanwhile the peak at 604 cm^−1^ represented the stretching vibration Fe–O bond in the material. FTIR spectra of chitosan shows peaks at 968, 2006, and 2975 cm^−1^ corresponded to the characteristic stretching vibrations of C–O, NH_2_, and C–H bonds, respectively. Meanwhile, FTIR spectra of PF127 showed peaks at 3500, 2800, and 1108 cm^−1^, correspond to O-H, C-H and C-O group stretching, respectively. FTIR spectra of α-mangosteen showed peaks at 3400, 2930, 1685, 1410, 1360 and 1027 correspond to O-H stretching, C-H stretching, C=O stretching, CH_2_ bending, CH_3_ bending and C-O ester, respectively. 

The FTIR spectra of the nanocomposites (Figure 5b) showed a synergistic effect between MRGO, chitosan, PF 127 and α-mangosteen. The peak at around 3300–3500 cm^−1^ appears in the Chi@MRGO, PF127-Chi@MRGO, and α-mangosteen-loaded PF127-Chi@MRGO spectra was correspond to the hydroxyl groups present in the adsorbed water molecules present at the surface of nanocomposites. The shifting FTIR peaks of α-mangosteen-loaded PF127-Chi@MRGO in compared with MRGO, Chi@MRGO, PF127-Chi@MRGO indicated the potential interaction between chitosan and PF 127 with MRGO.

**Figure 6:**
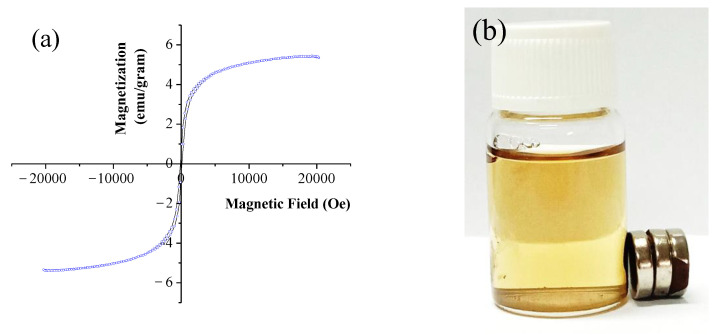
VSM of the α-mangosteen-loaded PF127-Chi@MRGO nanocomposites (**a**); the nanocomposites under magnetic field exposure (**b**).

Figure 6a shows the magnetization of α-mangosteen-loaded PF127-Chi@MRGO nanocomposites around 5 emu/gram. According to previous publications, the reported magnetic saturation value was low in comparison to that of Fe_3_O_4_ NPs [11]. This might be due to the presence of a high amount of non-magnetic polymer including PEG, chitosan and PF127 on the surface of GO. The prepared composite showed excellent response in the external magnetic field. Figure 6b shows that the nanocomposites could dispersed homogeneously without the presence external magnetic field and could attract easily with external magnetic field exposure confirming superparamagnetic characteristics. Previous study by Viswanathan et al. prepared Chitosan overlaid Fe_3_O_4_/reduced graphene oxide nanocomposite with the superparamagnetic characteristics with a saturation magnetization (MS) of 5.27 emu/g [36]. Eventually, the nanocomposites have the potential to be developed into a targeted drug delivery system and/or targeted nanomedicine for the treatment of cancer in the near future due to its excellent superparamagnetic characteristics, high colloidal stability, and dispersibility.

### 3.3. Stability of Nanocomposites

**Figure 7:**
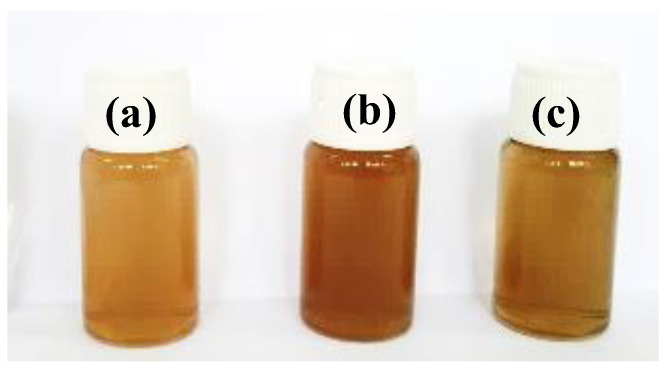
Stability of Chi@MRGO (**a**); PF127-Chi@MRGO (**b**); α-mangosteen-loaded PF127-Chi@MRGO (**c**).

Another important factor for targeted drug delivery applications is a nanomaterial’s dispersibility in water and physiological fluid. The dispersibility of Chi@MRGO and PF127-Chi@MRGO and α-mangosteen -loaded PF127-Chi@MRGO, is shown in Figure 7. 0.5 mg/mL of Chi@MRGO and PF127-Chi@MRGO and α-mangosteen-loaded PF127-Chi@MRGO were dispersed homogeneously. We observed the stability of MRGO after modification with chitosan and PF-127. We observed that the nanocomposites also show good stability and dispersibility. This result might be due to surface modification of chitosan that provided a positive charge on the surface of MRGO.

### 3.4. Encapsulation Efficiency of α-Mangosteen on PF127-Chitosan @MRGO

An encapsulation efficiency of α-mangosteen on drug carrier was 71.9%. α-mangosteen loading on the PF127-Chi@MRGO can occur via one of two mechanisms. Firstly, α-mangosteen loading on the reduced graphene oxide plane by π-π stacking and hydrogen bonding, or secondly, α-mangosteen loading on the PF127-Chi@MRGO via hydrophobic contact. 

### 3.5. In Vitro Cytotoxicity Study

**Figure 8:**
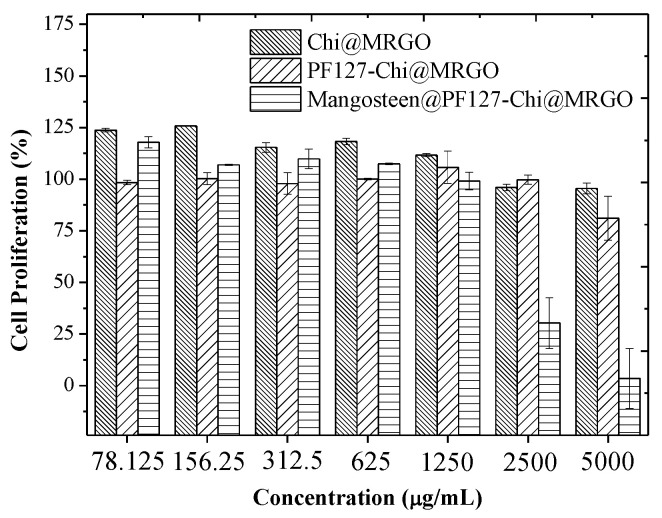
Cytotoxicity of Chi@MRGO, PF127-Chi@MRGO, and α-mangosteen-loaded PF127-Chi@MRGO nanocomposites against MCF-7 cell lines.

Compatibility and safety issue plays a crucial role in discovery and development of nanocomposites for drug carrier agent. In vitro cytotoxicity assay of drug carrier including Chi@MRGO, PF127-Chi@MRGO and α-mangosteen-loaded PF127-Chi@MRGO was evaluated at a various concentration range from 78.125 μg mL^−1^ until 5000 μg mL^−1^ through a resazurin-based cytotoxicity assay against MCF-7 cell lines (Figure 8). 

**Figure 9:**
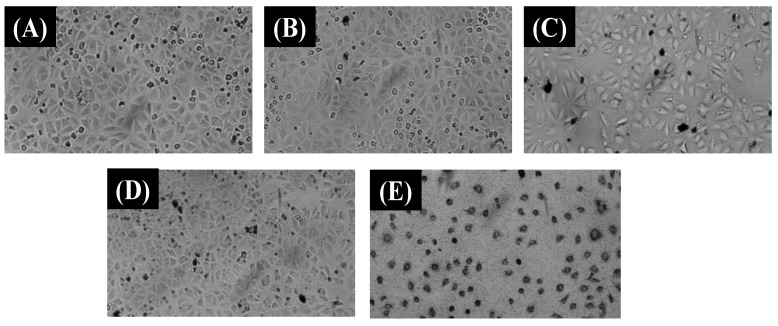
MCF-7 cell lines cellular image representation in DMEM without nanocomposites (**A**); after incubation with MRGO (312.5 μg/mL) (**B**); Chi@MRGO (5000 μg/mL) (**C**); PF127-Chi@MRGO (1250 μg/mL) (**D**); α-mangosteen-loaded PF127-Chi@MRGO (5000 μg/mL) (**E**).

Firstly, we investigated the toxicity of MRGO against MCF-7 cell lines. We observed that the MCF-7 cell lines proliferate indicating that the drug carrier precursor exhibits no cytotoxicity compared to the control sample and MRGO (312.5 μg/mL) in the cellular image representation in Figure 9A and 9B, respectively. Further, we investigated the influence of chitosan and PF127 in Chi@MRGO and PF127-Chi@MRGO as a drug carrier against cytotoxicity of MFC-7 cell lines using the drug carrier concentration ranging from 78.125, 156.25, 312.5, 625, 1250, 2500 and 5000 μg mL^−1^. Generally, we found that encapsulation of chitosan and PF127 into MRGO presence no cytotoxicity especially for the concentration ranging from 78.125 until 1250 μg mL^−1^ as the cellular image representation in Figure 9C and 9D for Chi@MRGO (5000 μg/mL) and PF127-Chi@MRGO (1250 μg/mL), respectively. This result is in good agreement with the previous investigation by Lee et al. that investigated the increasing biocompatibility and reducing toxicity of RGO-Fe_3_O_4_ after modification with PF127 on MRC-5 and A549 cell lines [15]. Meanwhile, at higher concentration of 2500 and 5000 μg mL^−1^, the drug carriers exhibit slightly minor growth inhibition. These results can be explained by the polysaccharides in chitosan could also exhibit anti-cancer capabilities and can stop the development of malignant cells. Eventually, our results confirmed that our drug carriers have no toxicity against cellular compartment. Furthermore, we investigated the toxicity of α-mangosteen in α-mangosteen-loaded PF127-Chi@MRGO. The result shows that α-mangosteen-loaded PF127-Chi@MRGO exhibits high toxicity into MCF-7 cell lines especially for the concentration of 2500 and 5000 μg mL^−1^ (Figure 9E). As a result, loading α-mangosteen onto PF127-Chi@MRGO increased the cytotoxicity of α-mangosteen against MCF-7 cell lines. 

Table 1 shows the various materials that have been used with α-mangosteen including fiber and biopolymer against various cancerous cells.

Previous study reported that the cancer growth and proliferation can be inhibited by α-mangosteen at all stages including cell division, proliferation, apoptosis, inflammation, and metastasis. The enzymes kinases, cyclooxygenase, ribonucleotide reductase, and DNA polymerase are all inhibited by α-mangosteen in tumor cells. Moreover, according to the in vitro and in vivo studies, α-mangosteen inhibits various tumor cell proliferations via changing a variety of targets and signal transduction pathways. When employed against cancer cells, α-mangosteen is more potent and focused. An exposure-induced cytotoxic antitumor response may lead to tumor cell death. Necrosis, autophagy, and apoptosis are three different ways that cells can death [6].

## 4. Conclusions

PF127-Chi@MRGO nanocomposites loaded with α-mangosteen as a drug carrier moiety have been successfully developed. FESEM observation shows the nanocomposites consist of magnetic nanoparticles tethered on the surface of PF-127-Chi@MRGO. The nanocomposite shows a good dispersion in an aqueous system and response to the external magnetic field exposure. Resazurin-based cytotoxicity assay confirmed that α-mangosteen-loaded PF127-Chi@MRGO could reduce the proliferation of MCF-7 cells in a concentration-dependent manner. Thus, the α-mangosteen-loaded PF127-Chi@MRGO can be used as a promising drug carrier for cancer treatment through combination of natural chemotherapy and biocompatible materials for the targeted-drug delivery system.

## Figures and Tables

**Table 1:** Recent nanoparticle formulation of mangosteen for drug delivery systems (DSSs).

No	Material	Mangosteen	Main Objective	Ref.
1	Nanocellulosic fibers (*Acetobacter xylinum)*	α-Mangosteen	multifunctional nanofiber films with antimicrobial and anticancer properties	[37]
2	Chitosan-Kappa Carrageenan	α-Mangosteen	improve cytotoxicity as breast cancer therapy agents	[38]
3	Poly (D, L-lactic-co-glycolic acid) (PLGA)	α-Mangosteen	inhibit colorectal cancer growth	[39]
4	Dioleoylphosphatidylcholine (DOPC), cholesterol, and polycarbonate membrane	α-Mangosteen	effective cytotoxic effect against human hepatoma Hep-G2 cells	[5]
5	Crosslinked silk fibroin-based nanoparticles using EDC or PEI as a crosslinker	α-Mangosteen	high potential for cancer chemotherapy	[33]
6	Monomethoxy poly (ethylene glycol)-polycaprolactones (MPEG-PCLs)	α-Mangosteen	inhibit the proliferation of melanoma cell and improve chemotherapeutic agent in melanoma therapy	[40]
7	Cyclodextrin-based nanoparticles	α-Mangosteen	Potential carrier for cancer therapy	[32]
8	β-cyclodextrin	α-Mangosteen	Improve bioavailability and maintain lung cancer cells activity	[41]
9	Chitosan/alginate using genipin as crosslinker	α-Mangosteen	antitumour activity to colorectal adenocarcinoma cells	[42]
10	Poly(ethylene glycol)–poly(l-lactide) (PEG–PLA)	α-Mangosteen	improve the effect of chemotherapy on pancreatic ductal adenocarcinoma (PDAC)	[43]
11	α-mangosteen -loaded PF127-Chi@MRGO nanocomposites	α-Mangosteen	Inhibit the proliferation of MCF-7 cells	This work

## Data Availability

The data presented in this study are available on request from the corresponding author.
